# An analysis of problem gambling among the Finnish working-age population: a population survey

**DOI:** 10.1186/1471-2458-13-519

**Published:** 2013-05-29

**Authors:** Sari Castrén, Syaron Basnet, Maiju Pankakoski, Jenni-Emilia Ronkainen, Satu Helakorpi, Antti Uutela, Hannu Alho, Tuuli Lahti

**Affiliations:** 1 Department of Mental Health and Substance Abuse Services, National Institute for Health and Welfare, Helsinki, Finland; 2 Faculty of Behavioural Sciences, Institute of Behavioural Sciences, University of Helsinki, Helsinki, Finland; 3 Faculty of Social Sciences, Department of Behavioural Sciences and Philosophy, University of Turku, Turku, Finland; 4 National Institute for Health and Welfare, Department of Lifestyle and Participation, Health Behaviour and Health Promotion, Helsinki, Finland; 5 School of Health Sciences, University of Tampere, Tampere, Finland

## Abstract

**Background:**

Gambling problems currently affect approximately 100 000 Finns. In order to prevent and reduce gambling-related harms it is crucial for the Finnish public health authorities to gain a stronger understanding of the association between gambling problems and related socio-demographic factors, other commonly co-occurring dependencies (e.g. alcohol and nicotine) and the type of games gambled. In this article the prevalence of problem gambling in Finland and the socio-demographic profiles of problem gamblers are studied.

**Method:**

An annual postal survey entitled Health Behaviour and Health among the Finnish Adult Population AVTK was sent to a random sample of Finnish adults (N=5000) aged between 15 and 64. The sample was derived from the Finnish Population Register. The survey was mailed to the participants in April 2010. Gender differences in socio-demographic variables and Problem Gambling Severity Index PGSI were assessed. A multinomial regression model was created in order to explore the association between socio-demographic factors and the severity of gambling.

**Results:**

A total of 2826 individuals (1243 males and 1583 females) replied to the survey. Of the respondents, 1.1% (2.1% of males, 0.3% of females) were identified as problem gamblers. Those who were of younger age, gender, had less than twelve years of education, consumed alcohol at risk level and smoked had higher odds of having low or moderate levels of gambling problems. Whereas, unemployment and smoking predicted significantly for problem gambling. Females gambled Lotto and slot machines less frequently than males and had more low level gambling problems. Males gambled more with a higher frequency and had a more severe level of gambling problems. Females were more attracted to scratch card gambling and daily Keno lotteries compared to males. In comparison, males gambled more on internet poker sites than females. Overall, a high frequency of gambling in Lotto, daily lotteries, slot machines, horse race betting and internet gambling was significantly associated with a more severe level of problem gambling.

**Conclusion:**

Gambling problems affect tens of thousands of individuals annually, therefore certain vulnerabilities should be noted. Comorbid dependencies, smoking in particular, ought to be screened for and recognised in the public health sector. Regulating the availability of slot machine gambling and enforcement of the age limit should be acknowledged. In establishing new gambling venues, prevalence rates in those particular areas should be actively monitored.

## Background

Many people gamble as a leisure activity, though for some it turns out not to be a leisure with pleasure. Gambling problems cause severe negative consequences. A DSM-IV (Diagnostic and Statistical Manual of Mental Disorders) diagnosis for pathological gambling (PG) requires ‘persistent and maladaptive gambling behaviour’, as indicated by at least five of ten symptoms that are similar in content to the symptoms of substance abuse [1]. Problem gambling is a milder form of gambling and is indicated by at least three of the ten DSM-IV-TR diagnostic criteria [2]. Recent analysis by Williams, Volberg and Stevens [3] concluded that the standardized past- year problem gambling [1] prevalence ranged from 0.5% to 7.6% with the average rate across all countries being 2.3%. According to their comprehensive report the lowest prevalence rates for problem gambling are in Europe, intermediate rates in North America and Australia and the highest in Asia. The current problem gambling^1^ rate in Finland is 2.7% [4].

To date there have only been three Finnish Gambling Prevalence Surveys [4-6]. The latest survey from 2011 revealed that 78% of the Finns had gambled over the past year, the most popular gambling activity being National Lottery (i.e. Lotto) with more than half of the Finnish population having gambled Lotto (74%). The next most popular game was scratch cards (37%) followed by slot machine gambling. Internet games (national and international) attracted 1% of the Finns.

A vast body of research has found that certain socio-demographic characteristics are associated with the development of gambling problems. Explicitly, younger age, male gender and socio-economic status (e.g. lower level of education, unemployment, marital status) are associated with gambling problems [7-11]. 

Gambling problems often co-occur with substance abuse and nicotine dependence. Lorains et al. [12] meta-analysis of comorbid disorders in pathological and problem gambling revealed that the weighted mean effect size for substance use disorders was 57.%, for alcohol use disorder 17.2% and 60.1% for nicotine dependence.

The availability and acceptability of legalized gambling has increased expansively over the past decades as noted by Lyk-Jensen [13]. This development has the potential to increase the prevalence of gambling problems. In addition to the availability of games, the types of games gambled also influence the development of gambling problems [14]. Globally, the utmost problems are associated with EMG’s, known as slot machines [14,15]. The same trend is seen in Finland: Finnish Gambling Clinic’s and Finnish Gambling Help Line – Peluuri’s annual reports [16] show that slot machine gambling is the most troubling type of game for help-seeking problem gamblers. In Finland there are unique opportunities to gamble, as slot machines, about 20 000 units, are freely dispersed in kiosks, restaurants, grocery stores, fuel stations and shopping centres. Availability, accessibility and acceptability of gambling is most likely to have a great impact on the overall gambling prevalence of the Finns.

Our aims were to investigate the prevalence of problem gambling in the adult sample in Finland, and to describe socio-demographic characteristics, alcohol consumption and nicotine dependency on different severity levels of gambling, and to also investigate types and frequency of games gambled among the adult population.

## Methods

### Recruitment

During April to June 2010, a total of 2826 individuals (1243 males and 1583 females) replied to the survey. An annual postal survey, entitled Health Behaviour and Health among the Finnish Adult Population (AVTK), was sent to a random sample of Finnish adults (N=5000) aged between 15 and 64. The sample was derived from the Finnish Population Register. The survey was mailed to the participants in April 2010. A total of three reminders were sent until June if the participants did not return the survey. Participants sent their replies by pre-paid mail. The primary purpose of the AVTK survey was to obtain information about current health-related behaviours of working-age Finns, and about long- and short-term changes in health-related behaviours among this population. The survey examined key aspects of health-related behaviours including: smoking, dietary habits, alcohol consumption and physical activity. Two sections of gambling-related questions were included in the survey. (Finnish report: http://urn.fi/URN:NBN:fi-fe201205085393).

Note: In 2008, for the first time in the history of the AVTK survey, gambling related-questions were included. Results of the year 2008 were published in Finnish [17]. The 2010 AVTK Health Survey results [18]. Analysis with gambling-related questions in 2010 presented here. The gambling-related questions of the 2011 [19] (in appropriate brackets) have not yet been analysed.

The study received ethics clearance from the Ethics Committee of the National Institute for Health and Welfare, Helsinki. Document number: THL/220/6.02.00/2010:§151/2010.

### Measures

For this study, we analysed the following sections of the AVTK survey: 1) Socio-demographic data, and 2) Finnish translation of the Problem Gambling Severity Index (PGSI), [20] where the sum of 9 items was computed, maximum points being 27, using a 4-point Likert scale with 0 = never, to 3 = almost always. Cronbach’s alpha was 0.79. The scoring of the PGSI is as follows: a) 0 = non-problem gambling, b) 1 or 2 = low level of gambling with few or no identified negative consequences (here considered to be low level gambling), c) 3 to 7 = moderate level of gambling leading to some negative consequences (here considered to be moderate level gambling) and d) 8 or more = problem gambling with negative consequences and a possible loss of control (here considered to be problem gambling). 3) The type of gambling was assessed by presenting 10 main types of gambling and frequency of gambling. The participants were asked to choose on what type of gambling they gambled. Gambling types were: a) Lotto and Viking lotto, b) daily Keno lotteries, c) slot machines, d) scratch cards, e) sports betting, f) horse race betting and g) internet poker via both PAF (Åland Slot Machine Association) and other international internet gambling sites. Frequency of gambling was measured using a 5-point Likert scale: not at all, less than once a week, 1–2 days per week, 3–5 days per week, 6–7 days per week. For gender and general comparisons responses were classified into two classes with regards to the frequency of gambling: less than once a week, and at least once a week. 4) Two questions of alcohol use: a) overall alcohol consumption: ‘During the past 12 months, have you consumed any alcohol?’ Yes/No answers, b) risk-level of alcohol consumption: ‘How often do you drink six or more units of alcohol?’ (One unit: 1/3 litre beer or cider, 12 cl wine, 8 cl strong wine, 4 cl strong alcohol), with a 6-point Likert scale where 1 = daily, 2 = 2–3 times per week, 3 = once a week, 4 = 2–3 times per month, 5 = couple of times per year or less, 6 = never. Risk-level alcohol consumption is defined as at least 6 units once a week. (Only question b) was used in the analyses, being a more accurate variable). 5) Nicotine dependency by asking smoking frequency: ‘Do you smoke at the moment (cigarettes, pipe or cigars)?’ with a 3-point Likert scale where 1 = yes, daily, 2 = once in a while, 3 = not at all.

### Participants

There were more females (56%) than males in this sample. The mean age of the respondents was 42.9 years (SD = 14.4). 36.6% of respondents were 51–65 years old, 24.2% were 26–40 years old, 22.5% were 41–50 years old and 16.4% were 16–25 years old. With regard to marital status, 65.3% of the respondents were married, 24.5% single, 8.5% divorced or separated and 1.3% widowed. The employment status of respondents was as follows: 62.1% employed, 2.4% partially employed or retired, 0.5% laid off, 5.7% unemployed, 13.7% students, 3.3% homemakers (stay-at-home mother or father), 0.7% on sick leave, 10.5% pensioned and 0.9% unemployed for any other reason. The response rate was 57%.

### Statistics

Gender differences in socio-demographic factors, frequency of gambling and PGSI were assessed using t-tests for continuous data and Chi square tests for categorical data. A multinomial regression model was created to explore the association between socio-demographic variables and the level of gambling severity (PGSI). Different severity levels of gambling were compared to the non-problem gambling group which served as the reference category. The statistical program SPSS (version 18) was used for the analyses.

## Results

### Socio-demographic characteristics and gender differences of the participants

#### Age and gender

The age difference between females (M = 42.3, SD = 14.4) and males (M = 43.6, SD = 14.32) was small but statistically significant (t (2824) = 2.40, p = 0.017). However, because of the large sample size, these kind of small and trivial differences often appear to be significant.

#### Marital status

As many as 60% of the respondents reporting the most severe forms of gambling problems were separated or divorced (χ2(9, 2727) = 24.1, p = 0.004). The severity of gambling problems was also compared with marital status, with 67.3% of those respondents with no gambling problems being married or cohabiting. Single status respondents had the highest percentage in both the low (17.5%) and moderate levels (6.9%) of gambling problems.

#### Education and employment

Females were significantly more educated than males in this sample (χ2 (1, 2727) = 52.94, p < 0.001) (Table 1). With regards to unemployment there were no significant differences between men and women (χ2 (1, 2821) = 0.87, p = 0.35) (Table 1).

**Table 1:** Gender differences in education, employment, alcohol consumption and smoking

**Measure**	**Gender**				**Chi-square test**
	**F**		**M**		
	**N**	**(%)**	**N**	**(%)**	
**Education** (yrs)					χ2 (1, 2784) = 52.94, p < 0.001
< 12 yrs	595	(38.2)	639	(52.0)	
> 12 yrs	961	(61.8)	589	(48.0)	
**Employment**					χ2 (1, 2821) = 0.87, p = 0.35
Employed	1495	(94.6)	1164	(93.8)	
Unemployed	85	(5.4)	77	(6.2)	
**Alcohol consumption**^**a**^					χ2 (1, 2760) = 138.15, p < 0.001
Non-risk level	1426	(92.0)	918	(75.9)	
Risk level	124	(8.0)	292	(24.1)	
**Nicotine dependency (smoking)**					χ2 (1, 2789) = 24.20, p < 0.001
Daily	248	(15.9)	288	(23.3)	
Occasionally / not at all	1307	(84.1)	946	(76.7)	

^a^ Risk-level alcohol consumption is defined as consuming at least 6 units of alcohol at least once a week.

#### Comorbid alcohol consumption and nicotine dependency (smoking)

Risk-level alcohol consumption was greater among males compared to females (χ2 (1, 2760) = 138.15, p < 0.001). Gender differences in smoking were also significant, indicating that males smoked more than females (χ2 (1, 2789) = 24.20, p < 0.001) (Table 1).

#### Prevalence and gender differences in severity of gambling

Of all respondents, a total of 1.1% were problem gamblers (8 or more points on the PGSI scale), with 5.5% of the respondents experiencing moderate levels of gambling problems. According to our results, males suffered from more severe forms of problem gambling than females. Specifically, gender differences in all three PGSI categories were significant as follows: for low level (males = 88.9%, females = 97%), for moderate level (males = 9.0%, females = 2.6%) and for problem gambling level (males = 2.1%, females = 0.3%) (χ2 (2, 2738) = 73.47, p < 0.001) (Table 2).

**Table 2:** Gender differences in severity level of gambling

**Gender**	**PGSI level**					**Chi-square test**
	**No problem**	**Low**	**Moderate**	**Problem gambling**	**Total**	
	**N (%)**	**N (%)**	**N (%)**	**N (%)**	**N (%)**	
Male	836 (68.6)	248 (20.3)	110 (9.0)	25 (2.1)	1219 (44,5)	χ2 (3,2738) = 154.24, p < 0.001
Female	1329 (87.5)	145 (9.5)	40 (2.6)	5 (0.3)	1519 (55.5)	
Total	2165 (79.1)	393 (14.4)	150 (5.5)	30 (1.1)	2738 (100)	

#### Type of games and the frequencies gambled

The most common form of gambling was lotto, reported to have been played by 56.4% of the respondents. Other popular game types were scratch cards (25.9%) and slot machine gambling (23.8%). Scratch card gambling was the only game type which was more popular among females (27.3%) than males (24.0%). In comparison, males favoured game types such as sports betting (14.1% males, 1.5% females), horse race betting (5.7% males, 1.7% females) and internet poker sites (5.4% males, 0.5% females).

Most of the game types were more frequently gambled by men compared to women. When examining only people who reported at least some degree of gambling activity, differences in the frequency of gambling exist in the following game types: lotto (χ2 (1576, 1) = 21.5, *p* < 0.001), slot machines (χ2 (649, 1) = 11.3, p = 0.001), sports betting (χ2 (190, 1) = 7.5, p = 0.006) and horse race betting (χ2 (94, 1) = 5.0, p = 0.03).

#### Frequency of gambling and the severity level of gambling

Table 3 shows types of gambling and the frequency of each game type gambled, less than once a week and at least once a week, within different levels of PGSI. Only subjects who reported at least some amount of gambling are included in the results. Type of games gambled are presented from highest to lowest frequencies.

**Table 3:** Type of games, frequency of gambling and the severity level of gambling

**Type of game**	**Frequency**	**PGSI level**^**a**^			**Total**
		**Low or no problem**	**Moderate**	**Problem gambling**	
		**N (%)**	**N (%)**	**N (%)**	**N (%)**
1. Lotto***	< once a week	795 (55.3)	36 (34.3)	6 (26.1)	837 (53.5)
	≥ once a week	642 (44.7)	69 (65.7)	17 (73.9)	728 (46.5)
	Total	1437 (100)	105 (100)	23 (100)	1565 (100)
2. Daily lotteries***	< once a week	188 (58.9)	18 (32.1)	4 (25.0)	210 (53.7)
	≥ once a week	131 (41.1)	38 (67.9)	12 (75.0)	181 (46.3)
	Total	319 (100)	56 (100)	16 (100)	391 (100)
3.Slot machine***	< once a week	412 (79.4)	50 (47.6)	11 (47.8)	473 (73.1)
	≥ once a week	107 (20.6)	55 (52.4)	12 (52.2)	174 (26.9)
	Total	519 (100)	105 (100)	23 (100)	647 (100)
4. Scratch cards#	< once a week	581 (92.7)	58 (87.9)	10 (83.3)	649 (92.1)
	≥ once a week	46 (7.3)	8 (12.1)	2 (16.7)	56 (7.9)
	Total	627 (100)	66 (100)	12 (100)	705 (100)
5. Sports Betting#	< once a week	103 (74.1)	24 (72.2)	9 (50)	136 (71.6)
	≥ once a week	36 (25.9)	9 (27.3)	9 (50)	54 (28.4)
	Total	139 (100)	33 (100)	18 (100)	190 (100)
6. Horse race Betting*	< once a week	49 (75.4)	10 (52.6)	4 (40.0)	63 (67.0)
	≥ once a week	16 (24.6)	9 (47.4)	6 (60.0)	31 (33.0)
	Total	65 (100)	19 (100)	10 (100)	94 (100)
7. Internet gambling**	< once a week	35 (81.4)	10 (47.6)	3 (37.5)	48 (66.7)
	≥ once a week	8 (16.8)	11 (52.4)	5 (62.5)	24 (33.3)
	Total	43 (100)	21 (100)	8 (100)	72 (100)

Note: Not Significant: #; Significant: *<0.05, **<0.01, ***<0.001.

^a^ No problem gambling included in low level of gambling problems.

Only subjects who reported at least some amount of gambling were included in the table.

#### Lotto and daily lotteries

Lotto was the most frequently gambled game in this sample with 53.5% of the respondents having gambled lotto less than once a week, and 46.5% gambled at least once a week. Daily lotteries were gambled by 53.7% of repondents less than once a week, and 46.3% gambled at least once a week. The frequency of lotto and daily lotteries betting was associated with gambling severity (χ2 (2, 1565) = 24.4, p <0.001 and (χ2 (2, 391) = 19.57, p < 0.001). That is, subjects with more severe gambling problems gambled these games more frequently compared to those with only low level problems or no gambling problems.

#### Slot machine gambling

Slot machine gambling attracted 26.9% of the respondents to gamble at least once a week and 73.1% gambled it less than once a week. Frequent slot machine gambling was associated with more severe gambling problems (χ2 (2, 647) = 52.57, p < 0.001).

#### Scratch cards

Scratch cards attracted 7.9% of respondents at least once a week and 92.1% gambled it less than once a week. The frequency of scratch card gambling was not associated with gambling severity (χ2 (2, 705) = 3.14, p = 0.24).

#### Sports betting

Of the respondents, 28% bet on sports at least once a week. The frequency of sports betting was not associated with gambling severity groups (χ2 (2,190) = 4.58, p = 0.10).

#### Horse race betting

Horse race betting was gambled by 33.0% of respondents with at least once a week frequency. Frequent horse race betting was associated with more severe gambling problems (χ2 (2, 94) = 7.14, p = 0.03).

#### Internet gambling

Internet gambling was gambled by 33.3% of respondents with at least once a week frequency. Frequent internet betting was associated with more severe gambling problems (χ2 (2, 72) = 10.7, p = 0.005).

#### Association between socio-demographic characteristics and levels of gambling severity

The multinomial regression model (Table 4) shows the association between socio-demographic variables and levels of gambling severity. Covariates in the model were age, gender, years of education, unemployment, risk-level alcohol consumption and daily smoking. Younger age was significantly associated with all levels of problematic gambling. Male gender was similarly recognized to be strongly associated with all problem gambling levels. Education (less than twelve years) was also found to be significantly associated with both a low level of problem gambling and even more strongly with a moderate level of problem gambling. Unemployment was most strongly associated with problem gambling. Risk-level alcohol consumption (at least 6 units at least once a week) was significantly associated with low and moderate gambling problems. Smoking had a strong and significant association with all levels of gambling problems (daily smoking was compared with occasional- and non-smoking).

**Table 4:** Multinomial regression analysis of variables associated with problem gambling severity

**Measures**	**Low level of problems**		**Moderate level of problems**		**Problem gambling**	
	**OR**	**95% CI**	**OR**	**95% CI**	**OR**	**95% CI**
Age^a^	0.98***	(0.97-0.99)	0.98**	(0.97-0.99)	0.97*	(0.94-0.99)
Male gender	2.46***	(1.94-3.12)	3.91***	(2.62-5.83)	7.51***	(2.78-20.29)
Education (<12yrs)	1.28*	(1.02-1.61)	1.95***	(1.36-2.81)	1.23	(0.56-2.69)
Unemployed	1.15	(0.72-1.83)	1.25	(0.64-2.44)	4.78**	(1.89-12.07)
Risk-level alcohol consumption^b^	1.62**	(1.21-2.16)	1.96**	(1.3-2.95)	0.74	(0.28-1.95)
Smoking (daily)	1.78***	(1.35-2.33)	1.80**	(1.21-2.68)	6.08***	(2.71-13.61)

Note. * p < .01 ** p < .001 *** p < .000.

Problem Gambling severity ( Reference group: non-problem gambling).

^a^Analysed as a continuous variable.

^b^ Risk-level alcohol consumption is defined as consuming at least 6 units at least once a week.

In summary, the significant associations for problem gambling were younger age, male gender, unemployment and daily smoking. Alcohol consumption, especially risk-level consumption, and lower years of education were significantly associated with both low and moderate gambling problems. According to the likelihood ratio test, the fit of the multinomial regression model was good (χ2 (18, 2826) = 275.9, p < 0.001). Correct classification rate was 79.2%.

## Discussion

### Socio-demographic characteristics

The analysis of socio-demographic characteristics shows that males were more vulnerable than females to develop gambling-related problems. This finding is in line with earlier studies both nationally [5-7,21] and internationally [22-24]. Additionally, young age was strongly associated with all levels of gambling. However, this association needs to be interpreted with caution, since even though the odds ratio was significant in the model, the strength of association was quite low. Divorced or separated individuals were also found to have more severe gambling problems compared to those married. Low level of education was associated with low and moderate levels of gambling problems and unemployment was associated with more severe gambling problems. These results resemble previous findings concerning socio-demographic characteristics of problem and pathological gamblers [7-11]. A vast body of evidence suggests that overall low socio-economic status is a risk factor for gambling, as also stated by Jimenez-Mucia et al. [25].

### Prevalence

The results from this study show that the prevalence of problem gambling is in line with the findings of Williams, Volberg and Stevens [3]. The prevalence rate in Finland falls in the average rates category among other European countries such as Sweden, Switzerland, Estonia and Italy. The findings of this study are also in line with previous studies from Finland indicating that the trend of problem gambling has been more or less unchanged during the past few years. There appears to be a declining trend based on Finnish Gambling Population Surveys [4-6] but it is not statistically significant according to the recent analysis by Raisamo and Salonen [26]. A unique finding of this study was that the moderate level of gambling was 5.5% in this sample. This is important to acknowledge, since moderate level gamblers may be at risk of developing gambling problems and so suffer from an increasing amount of negative consequences caused by gambling. Thus moderate levels of gambling need to be monitored closely in the future.

### Types of gambling

According to our results, Lotto was the most popular type of gambling, which has also been the case with previous population surveys in Finland [4]. Males bet on Lotto more frequently than females. On the other hand, females were more attracted to less risky gambling types such as lotto and scratch card gambling as compared to males also found by Hraba and Lee [27]. Frequent gambling in Lotto, daily lotteries, horse race betting and slot machine gambling were found to be associated with a more severe level of gambling.

In this study, slot machine gambling attracted the respondents gambling on a weekly basis. Slot machine gambling is classified as being addictive by nature [28] and has been reported to be the most trouble causing type of gambling among Finnish treatment-seeking gamblers [16]. The same trend was reported by Turja et al. [4] in a population sample where those who scored 5 or higher in SOGS reported their preferred game being slot machines. The rather high level of involvement in slot machine gambling, especially in Finland, could be explained by easy access and abundant availability. In Finland, slot machine gambling is easily and conveniently available in restaurants, grocery stores, shopping centres, kiosks and fuel stations around the country. A recent study in Finland by Warpenius et al. [29] investigated the enforcement of legal age limits on purchases of alcohol, tobacco and gambling slot machines. Their study showed that the enforcement of legal age limits for gambling slot machines was the weakest (4%) compared to purchases of alcohol (49%) and cigarette (43%). One of the reasons for the insufficient enforcement of the law is, at least in kiosks, shopping centres and fuel stations, that the locations of slot machines are often out of reach of the shop keepers’ desks, gambling time can be rather short and the gambler does not need to confront the shop keeper directly.

Availability, proximity and convenience of gambling venues have been found as being clear risk factors for gambling problems [30-32]. As a whole, males seem to choose riskier and faster game types, such as games like internet poker, which could partially explain why males tend to have more severe levels of gambling problems. Our results show that frequent involvement in internet gambling is associated with more severe levels of gambling problems. Our results also show that female moderate level of gambling was 2.6%, which is nearly three times lower compared to males. Females have been found to gamble often because of boredom, loneliness and isolation and thus maximize their gambling time with slower games [33,34]. Nevertheless, females have a growing risk in developing gambling problems, because progression in gambling problems is reported to be more rapid with females [35] and is known as telescoping phenomenon [36].

### Comorbid alcohol and nicotine dependency

In this sample, males consumed remarkably more alcohol than females. Risk-level alcohol consumption and male gender were strongly associated with low and moderate levels of gambling problems. Comorbidity with substance use, especially alcohol, has been frequently reported in earlier gambling studies. The rate of pathological gambling among substance abusers has been reported to be four to ten times greater when compared to the general population [37,38]. Similarly, the study by Hakkarainen et al. [21] from Finland stated that especially heavy episodic drinking increased the risk of problem gambling. Petry has reported that the odds ratio for any alcohol use disorder and alcohol dependence as well as drug use and smoking are significantly related to pathological gambling [39].

In fact, nicotine dependence “accounts for some elevated risks for psychopathology with subsyndromal and problem/pathological levels of gambling” and subsyndromal level of gambling are associated with more severe psychopathology, as stated by Grant, Desai and Potenza [40]. In our study, nicotine dependence was significantly associated with all levels of gambling severity. This is consistent with previous findings [37-39,41]. Nicotine dependence is the second most frequent addiction after alcohol use disorder. Moreover, Petry, Stinson and Grant [41] reported that nicotine dependent individuals had a seven times higher odds ratio to be pathological gamblers when compared to non-smokers. Additionally, women with nicotine dependence were 14 times more likely to be pathological gamblers when compared to non-smoking women. In contrast, the likelihood for nicotine dependent males being pathological gamblers was five times higher when compared to non-smokers. Petry and Oncken [42] found that daily smokers were less able to control their gambling and had more severe gambling problems when compared to non-smokers. The association between gambling problems and nicotine dependence is evident, and one dependence may serve as a prime for another, as suggested by McGrath and Barrett [43].

### Associations of socio-demographic characteristics, alcohol consumption, smoking and gambling severity

The results from this study revealed that younger age, male gender and daily smoking were associated with all levels of gambling problems. The notable likeness between low level and moderate level of gambling problems were less than twelve years of education and risk-level alcohol consumption. In contrast, unemployment was associated with problem gambling. These findings are soundly in line with previous findings and confirms that there appears to be certain groups of individuals that are more vulnerable to developing gambling problems.

### Limitations

Despite the large sample size and good representation of the Finnish population, there are a number of limitations with the data, possibly the self-administered survey being the most important. A self-administered survey is not as candid and honest as a face-to-face interview, and future studies would benefit from face-to-face interviews or correction weights (1.00) as suggested by Williams et al. [3]. Two previous studies have found a post-questionnaire to produce higher rates of prevalence compared to telephone interviews [3]. Another aspect to mention is the response rate of 57%, which is low compared to face-to-face interviews or telephone interviews, but still adequate [3].

Another limitation is that this study reflects its results with previous prevalence studies from Finland. Previous prevalence studies from Finland [4-6] have used the South Oaks Gambling Screen (SOGS) as a measure of gambling. This study uses Problem Gambling Severity Index (PGSI) which is more conservative than SOGS, because it uses 8 as a cut-off point as compared to SOGS that uses 5. This study’s gambling-related questions were part of a larger health survey. This means that this study’s prevalence rate ought to be more realistic compared to specific gambling surveys, because specific gambling surveys may attract gamblers more than health surveys and produce higher prevalence rates [3].

This study used PGSI which has not been validated in a Finnish cultural context. So far, there are no validated gambling scales available in Finland.

## Conclusions

To conclude, there is clear evidence that males seem to be more at risk of developing severe gambling problems. However, females should not be ignored because of two underlying factors: a) nicotine-dependent females are more vulnerable to developing gambling problems, b) development of gambling problems in females is faster as compared to males due to the telescoping phenomenon. Moreover, low socio-economic status, like low level of education and unemployment, can be seen as a risk factor for severe gambling problems.

The problem gambling prevalence in Finland has been more or less unchanged during past years. However, based on our findings it is as important to pay attention to the groups of low level and moderate level gamblers as their socio-demographic characteristics, and comorbid alcohol use and nicotine dependence all greatly resemble those of problem gamblers. These underlying factors linked with growing opportunities to gamble, especially in Finland, are only worsening the situation for those specific groups of individuals that may be at risk of shifting from one severity level to another.

From a public health perspective, these recognized associations should be taken into account by public health policy makers. Cox et al. [44] found that a high concentration of slot machines and the presence of a permanent casino were associated with an increased prevalence of gambling. This notion is important in the Finnish context with a visibly abundant amount of slot machines available, and a new casino complex emerging in the eastern part of Finland in the near future. Limitation of access to slot machines in Finland should be considered. The enforcement of the legal age limit on gambling should be made easier by limiting slot machines to the dedicated gambling area, where the enforcement of law is easier. The prevalence rate of gambling, in the eastern part of Finland, where the new casino complex emerges, should be closely monitored in the future. Gambling is a potential health issue, and there is a growing need to intensify awareness in the medical and health professions about gambling problems and related conditions. Awareness that three dependencies may co-occur, and should be screened for consistently in common health checks, especially within a vulnerable population.

### Endnote

^1^Problem gambling includes also Pathological Gambling.

## Competing interests

The authors declare that they have no competing interests.

## Authors’ contributions

SC, SB, MP, J-ER, SH, AU, HA, TL have contributed to the design of the study and interpretation of data. SC, SB, MP, SH, AU, HA, TL have been involved in drafting the manuscript or revising it critically for intellectual content. All authors read and approved the final manuscript.

## Pre-publication history

The pre-publication history for this paper can be accessed here:

http://www.biomedcentral.com/1471-2458/13/519/prepub

## References

[B1] American Psychiatric Association Diagnostic and statistical manual of mental disorders 1994 4 Washington DC: American Psychiatric Association

[B2] Rosenthal RJ Pathological gambling Psychiatr Analyses 1992 22 72 8

[B3] Williams RJ Volberg RA Stevens RMG The population Prevalence of Problem Gambling: Methodological Influences, Standardized Rates, Jurisdictional Differences, and Worldwide Trends 2012: Report prepared for the Ontario Problem Gambling Research Centre and the Ontario Ministry of Health and Long Term Care http://hdl.handle.net/10133/3068

[B4] Turja T Halme J Mervola M Järvinen-Tassopoulos J Ronkainen J-E Suomalaisten rahapelaaminen 2011 (Finnish gambling 2011) 2012 Helsinki: National Institute for Health and Welfare (THL)

[B5] Ilkas H Turja T Rahapelitutkimus. (Gambling study) 2003 Helsinki: Taloustutkimus Oy. Sosiaali ja terveysministerio (Ministry of Social Affairs and Health)

[B6] Aho P Turja T Suomalaisten rahapelaaminen 2007. (Finnish gambling 2007) Taloustutkimus Oy 2007 2007 Helsinki: Sosiaali ja terveysministerio (Ministry of Social Affairs and Health)

[B7] Johansson A Grant JE Kim SW Odlaug BL Götestam KG Risk factors for problematic gambling: A critical literature review J Gambl Stud 2009 25 67 92 10.1007/s10899-008-9088-6 18392670

[B8] Kessler RC Hwang I LaBrie RA Petukhova M Sampson N DSM-IV pathological gambling in the National Commorbidity Survey Replication Psychol Med 2008 38 351 60 10.1017/S0033291708002900 PMC2293303 18257941

[B9] Toneatto T Nguyen L Smith G, Hodgins DC, Williams RJ Individual characteristics and problem gambling behavior Research and measurements Issues in Gambling Studies 2007 San Diego, CA: Academic Press 279 203

[B10] Blanco C Hasin DS Petry N Stinson FS Grant BF Sex differences in subclinical and DSM-IV pathological gambling: Results from the National Epidemiologic Survey on Alcohol and Related Conditions Psychol Med 2006 36 943 53 10.1017/S0033291706007410 16650342

[B11] Welte J Barnes G Tidwell M Hoffman J The prevalence of problem gambling among U.S. adolescents and young adults: results from a national survey J Gambl Stud 2008 24 199 33 10.1007/s10899-007-9086-0 PMC4405098 18097737

[B12] Lorains FK Cowlishaw S Thomas S Prevalence of comorbid disorders in problem and pathological gambling: systematic review and meta-analysis of population surveys Addiction 2011 106 490 498 10.1111/j.1360-0443.2010.03300.x 21210880

[B13] Lyk-Jensen SV New evidence from the grey area: Danish results for at-risk gambling J Gambl Stud 2010 26 3 455 67 10.1007/s10899-009-9173-5 20066558

[B14] Griffiths MD Gambling technologies: Prospects for problem gambling J Gambl Stud 1999 15 265 283 10.1023/A:1023053630588 12766464

[B15] Parke J Griffiths MD The psychology of fruit machine: The role of structural characteristics (revisited) Int J Ment Health Addic 2006 4 151 79 10.1007/s11469-006-9014-z

[B16] Jaakkola T Murto A Pajula M Peliklinikan toimintakatsaus 2012 ja Peluurin puolivuotisraportti 2012 2012: Annual Report of the Gambling Clinic of Helsinki, 2012 and Half Yearly Report of Gamblers Helpline *Unpublished Finnish report 2012.* Retrieved from: http://www.peluuri.fi/data/liitteet/peliklinikka_toimintakatsaus_2012_pdf

[B17] Piispa M Laitilainen E Helakorpi S Halme J Alho H Uutela A Rahapelaaminen, pelaamisen aiheuttamat ongelmat ja niiden yhteys elintapoihin. Tutkimus työikäisistä suomalaisista vuonna 2008 (Gambling, gambling problems and their relation to living habits among Finnish adults in 2008) Helsinki: National Institute for Health and Welfare, Report 29/2009 http://www.thl.fi/thl-client/pdfs/295fc98c-8911-4656-88b0-4ec08da9645b

[B18] Helakorpi S Pajunen T Jallinoja P Virtanen S Uutela A Suomalaisen aikuisvaeston terveyskayttaytyminen ja terveys, kevat 2010 (Health Behaviour and Health among the Finnish Adult Population, Spring 2010) 2011 Helsinki: National Institute for Health and Welfare

[B19] Helakorpi S Holstila A-L Virtanen S Uutela A Suomalaisen aikuisvaeston terveyskayttaytyminen ja terveys, kevat 2011 (Health Behaviour and Health among the Finnish Adult Population, Spring 2011 2012 Helsinki: National Institute for Health and Welfare

[B20] Ferris J Wynne H The Canadian Problem Gambling Index: Final report 2001 Ottawa, Ontario: Canadian Centre on Substance Abuse

[B21] Hakkarainen P Jarvinen- Tassopoulos J Metso L Makela P, Mustonen H, Tigersted K Miten alkoholinkäyttö, rahapelaaminen ja huumeidenkäyttö kytkeytyvät toisiinsa? (How alcohol consumption, gambling ja drug use associate with each other?) in Suomi juo ( Title: Finland Drinking) 2010 Helsinki: National Institute for Health and Welfare. Yliopistopaino

[B22] Lejoyeux M McLoughlin, M, Ades J: Epidemiology of behavioural dependence: literature review and results of original studies Eur J Psychiatry 2000 15 129 34 10.1016/S0924-9338(00)00201-7 10881210

[B23] Potenza MN Steinberg MA McLaughlin SD Wu R Rounsaville BJ O’Malley SS Gender-related differences in the characteristics of problem gamblers using a gambling helpline Am J Psychiatry 2001 158 1500 1505 10.1176/appi.ajp.158.9.1500 11532738

[B24] Kessler C Hwang I LaBrie R Petukhova M Sampson NA Winters KC DSM-IV pathological gambling in the national comorbidity survey replication Psychol Med 2008 38 1351 1360 18257941 10.1017/S0033291708002900 PMC2293303

[B25] Jimenez-Murcia S Stinchfield R Fernandez-Aranda F Santamarıa JJ Penelo E Granero R Gomez-Pena M Aymamıa N Moragas L Soto A Menchon JM Are online pathological gamblers different from non-online pathological gamblers on demographics, gambling problem severity, psychopathology and personality characteristics? Int Gambl Stud 2011 11 325 337 10.1080/14459795.2011.628333

[B26] Raisamo S Salonen A Rahapelaaminen Suomessa vuosina 2003–2011. (Gambling in Finland in 2003–2011) 2013 Helsinki Unpublished manuscript

[B27] Hraba J Lee G Problem Gambling and Policy Advice: The Mutability and Relative Effects of Structural, Associational and Attitudinal Variables J Gambl Stud 1996 11 105 121 24233425 10.1007/BF02107110

[B28] Griffiths MD Wood RTA Parke J Parke A Gaming research and best practice: Gaming industry, social responsibility and academia Casino Gaming Int 2007 3 97 103

[B29] Warpenius K Holmila M Raitasalo K Peliin ei puututa. Alkoholin, tupakan ja rahapeliautomaattien ikärajavalvontaa testanneet ostokokeet vähittäisliikkeissä. (Enforcing age limits on purchases of alcohol and tobacco and the use of slot machines: test purchases in retail outlets) Yhteiskuntapolitiikka 2012 77 4 375 385

[B30] Moore SM Thomas AC Kyrios M Bates G Meredyth D Gambling accessibility: A Scale to Measure Gambling Preferences J Gambl Stud 2011 27 129 143 10.1007/s10899-010-9203-3 20526858

[B31] Breen H Risk and protective factors associated with gambling products and services: Indigenous gamblers in North Queensland Int J Mental Health Addict 2012 10 24 38 10.1007/s11469-010-9296-z

[B32] Brown S Coventry L Queen of hearts: The needs of women with gambling problems 1997 Melbourne, Australia: Financial and Consumer Rights Council

[B33] Trevorrow K Moore S The Association Between Loneliness, Social Isolation and Women's Electronic Gaming Machine Gambling J Gambl Stud 1998 14 263 84 10.1023/A:1022057609568

[B34] Ladd G Petry N Gender differences among pathological gamblers seeking treatment Exp Clin Psychopharmacol 2002 10 302 09 12233991 10.1037//1064-1297.10.3.302

[B35] Grant JE Potenza MN Weinstein A Gorelick DA Introduction to Behavioral Addictions Am J Drug Alcohol Abuse 2010 36 5 233 241 10.3109/00952990.2010.491884 20560821 PMC3164585

[B36] Lesieur HR Blume SB Zoppa RM Alcoholism drug abuse and gambling Alcohol Clin Exp Res 1986 10 33 38 10.1111/j.1530-0277.1986.tb05610.x 3515989

[B37] Spunt B Dupont I Lesieur H Liberty HJ Hunt D Pathological gambling and substance misuse: a review of the literature Subst Use Misuse 1998 33 2535 2560 10.3109/10826089809059340 9818989

[B38] Petry N Petry N Comorbidity of disordered gambling and other psychiatric disorders Pathological Gambling: Etiology, Comorbidity and Treatment 2005 Washington, DC: American Psychological Association

[B39] Grant JE Desai RA Potenza MN Relationship of Nicotine Dependence, Sybsyndromal and Patyhological Gambling, and Other Psychiatric Disorders: Data From the National Epidemiologic Survey on Alcohol and Related Conditions J Clin Psychiatry 2009 70 3 334 343 10.4088JJCP.08m04211 19254518 10.4088/jcp.08m04211 PMC3691098

[B40] Halme J Helakorpi S Laitalainen E Uutela A Alho H Rahapelaamisen tiheys on yhteydessä terveysriskeihin työikäisillä suomalaisilla. Gambling frequency is associated with health risks in Finnish working-age population Sosiaalilääketieteellinen aikakausilehti 2010 47 98 108

[B41] Petry NM Stinson FS Grant BF Comorbidity of DSM-IV pathological gambling and other psychiatric disorders: results from the National Epidemilogic Survey on Alcohol and Related Conditions J Clin Psychiatry 2005 66 564 74 10.4088/JCP.v66n0504 15889941

[B42] Perty NM Oncken C Cigarette smoking is associated with increased severity of gambling problems in treatment-seeking gamblers Addiction 2002 97 745 53 10.1046/j.1360-0443.2002.00163.x 12084144

[B43] McGrath DS Barrett SP The comorbidity of tobacco smoking and gambling: a review of the literature Drug Alcohol Rev 2009 28 676 81 10.1111/j.1465-3362.2009.00097.x 19930023

[B44] Cox BJ Yu N Afifi TO Ladouceur R A national survey of gambling problems in Canada Can J Psychiatry 2005 50 213 17 15898460 10.1177/070674370505000404

